# National Capacity for Surveillance, Prevention, and Control of West Nile Virus and Other Arbovirus Infections — United States, 2004 and 2012

**Published:** 2014-04-04

**Authors:** James L. Hadler, Dhara Patel, Kristy Bradley, James M. Hughes, Carina Blackmore, Paul Etkind, Lilly Kan, Jane Getchell, James Blumenstock, Jeffrey Engel

**Affiliations:** 1 Yale University School of Public Health; 2 Council of State and Territorial Epidemiologists, Atlanta, Georgia; 3 Oklahoma State Department of Health; 4 Emory University School of Medicine and Rollins School of Public Health, Atlanta, Georgia; 5 Florida Department of Health; 6 National Association of City and County Health Officials, Washington, DC; 7 Association of Public Health Laboratories, Silver Spring, Maryland; 8 Association of State and Territorial Health Officials, Arlington, Virginia

In the first 5 years after its introduction in the United States in 1999 (1), West Nile virus (WNV) spread to the 48 contiguous states, resulting in 667 reported deaths (1–3). To establish detection and response capacity, WNV surveillance and prevention was supported through CDC Epidemiology and Laboratory Capacity (ELC) cooperative agreements with all 50 states and six large cities/counties.* In 2005, the Council of State and Territorial Epidemiologists (CSTE) conducted an assessment of ELC recipients and determined that, since 1999, all had developed WNV surveillance and control programs, resulting in a national arboviral surveillance infrastructure (4). From 2004 to 2012, ELC funding for WNV surveillance decreased by 61%. In 2012, the United States had its most severe WNV season since 2003 (3), prompting a follow-up assessment of the capacity of ELC-supported WNV programs. Since the first assessment, 22% of jurisdictions had stopped conducting active human surveillance, 13% had stopped mosquito surveillance, 70% had reduced mosquito trapping and testing, and 64% had eliminated avian mortality surveillance. Reduction in early detection capacity compromises local and national ability to rapidly detect changes in WNV and other arboviral activity and to initiate prevention measures. Each jurisdiction is encouraged to review its current surveillance systems in light of the local threat of WNV and emerging arboviruses (e.g., dengue and chikungunya) and ensure it is able to rapidly detect and respond to critical changes in arbovirus activity.

Using the 2005 CSTE assessment procedure that measured capacity in 2004; new CDC guidelines for WNV surveillance, prevention, and control (5); and technical assistance from CDC, a CSTE workgroup developed an assessment tool to describe human, mosquito, and laboratory surveillance capacity for WNV and other arboviruses in 2012 and to compare responses with those from 2004. The workgroup included representation from the Association of State and Territorial Health Officers (ASTHO), the National Association of County and City Health Officials (NACCHO), and the Association of Public Health Laboratories (APHL). CSTE distributed the assessment form electronically in August 2013. Responses were received from all 50 states and all six ELC-supported city/county health departments.

## Surveillance Capacity

**FIGURE 1:**
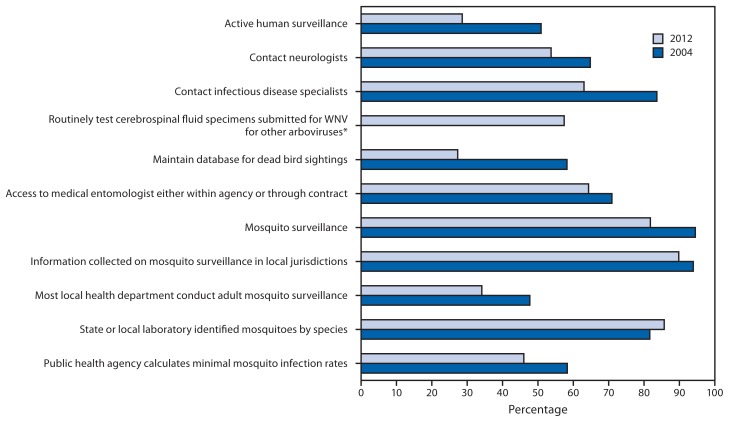
West Nile virus (WNV) surveillance capacity in state and Epidemiology and Laboratory Capacity–supported city/county health departments, by selected indicators — United States, 2012 and 2004 * Not assessed in 2004.

All 56 jurisdictions conducted surveillance for human WNV disease in 2012. Compared with 2004, they were less likely to have an active component to human surveillance (16 of 56 [29%] versus 28 of 55 [51%]) and were less likely to report contacting neurologists (29 of 54 [54%] versus 35 of 54 [65%]) or infectious disease specialists (34 of 54 [63%] versus 46 of 55 [84%]) by telephone, fax, mail, or electronic health alerts to encourage disease reporting (Figure 1). In 2012, 27 of 47 (57%) responding public health laboratories routinely tested human specimens submitted for WNV testing for other arboviruses, but of these, only six routinely tested for arboviruses other than St. Louis or eastern equine encephalitis viruses.

Mosquito surveillance capacity also decreased between 2004 and 2012. Fewer jurisdictions had their own mosquito surveillance systems (45 of 55 [82%] in 2012 versus 52 of 55 [95%] in 2004) and access to a medical entomologist either within the agency or through contract with another agency (36 of 56 [64%] versus 39 of 55 [71%]). Also decreasing were the number of states collecting information about mosquito surveillance in local health departments in their state (44 of 49 [88%] versus 46 of 49 [94%]), the number responding that ≥50% of local health departments in their state conduct adult mosquito surveillance (15 of 44 [34%] versus 21 of 44 [48%]), and the number of surveyed jurisdictions that calculated minimum mosquito infection rates (23 of 50 [46%] versus 28 of 48 [58%]). Only the number of jurisdictions that received information about the species of trapped mosquitoes increased (42 of 49 [86%] versus 40 of 49 [82%]) (Figure 1).

The assessment measured current staffing levels for WNV and other mosquito-borne virus surveillance in two ways: 1) the number of persons (direct hires or contractors) working as ≥50% full-time equivalents (FTEs) on WNV surveillance in the health department by funding source and 2) the total number of FTEs currently working by function (epidemiologist, laboratory staff, mosquito/other environmental surveillance, and “other”). The assessment also gathered information on additional staffing needs by function to be able to “achieve full epidemiology and laboratory capacity to conduct WNV and other mosquito-borne disease surveillance.”† Compared with 2004, the number of persons working as ≥50% FTEs on WNV in 2012 decreased 38%, from 382 to 235. Overall, 236.8 FTEs (including <50% FTEs) were working in the 56 jurisdictions at the time of the assessment, with 18% working as epidemiologists, 28% working in laboratory positions, 31% working on mosquito/environmental surveillance, and 24% working as support staff. Forty (80%) of the 50 states and four of the six local jurisdictions reported needing at least one additional FTE, for a total of 137.6 FTEs needed, 58% more than are currently employed (Table).

**FIGURE 2:**
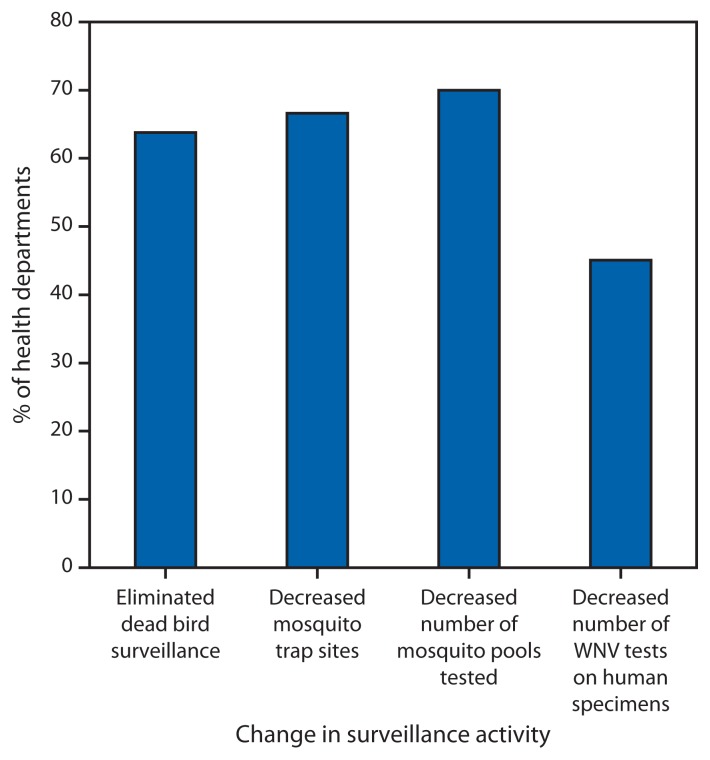
Percentage of Epidemiology and Laboratory Capacity (ELC)–funded state and city/county health departments modifying selected surveillance activities in the past 5 years in response to reduction in West Nile virus (WNV)–specific ELC funding, August 2013

Jurisdictions were asked how they had managed reductions to ELC funding for WNV surveillance during the past 5 years. Among respondents to specific questions, 30 of 47 (64%) eliminated dead bird surveillance, 32 of 48 (67%) decreased the number of mosquito trap sites, 35 of 50 (70%) decreased the number of mosquito pools tested, and 23 of 51 (45%) decreased the number of WNV tests done on human specimens (Figure 2). Jurisdictions identifying a need for additional laboratory staff were less likely than those with no additional need to test mosquito pools for WNV (25 of 33 [76%] versus 18 of 21 [86%]) and to test human cerebrospinal fluid specimens submitted for WNV testing for other arboviruses (15 of 28 [54%] versus 11 of 17 [65%]). They were more likely to have decreased the number of mosquito pools tested (22 of 32 [69%] versus 11 of 17 [65%]). Those identifying a need for additional mosquito surveillance staff were more likely than those without a need to have decreased the number of mosquito trapping sites (24 of 31 [77%] versus six of 15 [40%]).

## Prevention

In 2012, 51 of 56 (91%) jurisdictions posted prevention information about WNV on their websites compared with 54 of 55 (98%) in 2004. As of 2012, 33 of 53 (62%) jurisdictions had a formal plan for killing adult mosquitoes in the event of a WNV disease outbreak, and 15 of 47 (32%) states financially supported larviciding in at least some of their local health departments; at least another 14 would have supported larviciding if given sufficient funding.

### Discussion

Before the availability of WNV-specific ELC funding in 2000, no federal funding supported state and local arboviral surveillance, and no national arboviral surveillance infrastructure existed to respond to either introduced threats (e.g., WNV, dengue virus, and chikungunya virus) (6,7) or to potentially emerging endemic arboviruses (e.g., Powassan, LaCrosse, and Heartland viruses) (8). ArboNET, the national surveillance platform built to monitor WNV and expanded to include other arboviruses, is a distributed system dependent on each state and local health department having sufficient human, animal, and mosquito surveillance and reporting activities and supportive laboratory capacity to meet its prevention and control needs. Any change in state or local capacity affects both the local and national systems.

What is already known on this topic?

In response to the emergence of West Nile virus (WNV) in 1999, CDC Epidemiology and Laboratory (ELC) cooperative agreement funding supported surveillance and prevention capacity building in every state to detect and respond to WNV and other arboviruses. By 2004, every state had a high level of surveillance and prevention capacity, as measured by an assessment conducted by the Council of State and Territorial Epidemiologists (CSTE), and a national surveillance system based on state capacity was well established.

What is added by this report?

From 2004 to 2012, ELC cooperative agreement funding for arboviral surveillance decreased 61%. A recent CSTE assessment found that state and local health department capacity for WNV and other arbovirus surveillance and control have decreased substantially, and that some health departments had lost all mosquito monitoring capability and laboratory capacity to test for emerging arboviruses.

What are the implications for public health practice?

The loss of arboviral surveillance capacity might have compromised local and national ability to rapidly detect and respond to changes in WNV and other arboviral activity. Based on the findings in this assessment and current arboviral threats to the United States, jurisdictions are encouraged to review their current surveillance systems and ensure they meet with current CDC guidance and are able to rapidly detect and respond to critical changes in arbovirus activity.

The findings of the recent CSTE assessment demonstrate that critical state-level monitoring capacity built for WNV has eroded since 2004, despite states having largely eliminated less critical activities such as avian mortality surveillance. With states having cut back on mosquito surveillance, active surveillance for human disease, and laboratory testing for WNV and other arboviruses, the ability to rapidly detect emerging and outbreak-threshold threats and to rapidly initiate prevention measures to minimize human morbidity and mortality (e.g., public notification and killing adult mosquitoes) might be compromised. This comes at a time when the need for a robust early detection system is high: 2012 was one of the most intense WNV seasons since 1999, with 2,873 cases of neuroinvasive disease and 286 deaths reported. The threat of dengue outbreaks is growing, with an average of 492 imported cases detected in more than 30 states annually during 2010–2012 (9). In 2013, local dengue transmission was documented in Florida, Texas, and New York (9), and chikungunya virus transmission was documented in the Americas for the first time (10). Monitoring also serves to detect and track alterations in transmission ecology and epidemiology, including those that might occur as a result of climate change, and currently less common endemic arboviruses (8). Although the ELC funding language for WNV capacity building was expanded in 2005 to include other arboviruses, ELC funding for arbovirus surveillance has decreased.

The findings in this report are subject to at least two limitations. First, not all respondents answered all questions. Second, some respondents might have interpreted some questions differently in 2012 than in 2004.

This assessment focused on capacity to conduct currently recommended priority arbovirus surveillance functions that have been demonstrated to be of the most value in predicting outbreaks: surveillance for human disease, mosquito trapping and testing, and laboratory testing (5). Based on the findings in this assessment and current arboviral threats to the United States, jurisdictions are encouraged to review their current surveillance systems and ensure they meet with current CDC guidance and are able to rapidly detect and respond to critical changes in arbovirus activity.

## Figures and Tables

**TABLE:** Current staff working as full-time equivalents (FTEs) and additional staff needed to achieve full capacity for West Nile virus and other arboviral surveillance, by functional category — 50 states and six Epidemiology and Laboratory Capacity–funded city/county health departments,* August 2013

Functional category	2013 actual FTEs	Additional staff needed to achieve full capacity†	Increase needed (%)
Epidemiologist	41.5	28.1	(67.7)
Laboratory	66.5	29.4	(44.2)
Mosquito/Environmental	72.8	60.6	(83.2)
Other§	56.0	19.5	(34.8)
**Total**	**236.8**	**137.6**	**(58.1)**

* Chicago, Illinois; Houston, Texas; Los Angeles County, California; New York, New York; Philadelphia, Pennsylvania; and the District of Columbia.

† Defined as 1) ability to complete a standard case report form on every suspected/confirmed mosquito-borne arboviral disease case and report it to ArboNet, 2) ability to test by immunoglobulin M for all relevant arboviruses (including dengue) on any cerebrospinal fluid or serum specimen submitted to the state or city/county laboratory on a suspected case of arboviral disease), and 3) have an environmental surveillance system that includes mosquito surveillance to routinely monitor arboviral activity in larval and adult mosquitoes in all parts of the jurisdiction in which there is the potential for human outbreaks of arboviral disease based on past experience.

§ Other includes “other surveillance, clerical, and administrative staff.”

## Footnotes

* Chicago, Illinois; Houston, Texas; Los Angeles County, California; New York, New York; Philadelphia, Pennsylvania; and the District of Columbia.

† Defined as 1) ability to complete a standard case report form on every suspected/confirmed mosquito-borne arboviral disease case and report it to ArboNet, 2) ability to test by immunoglobulin M for all relevant arboviruses (including dengue) on any cerebrospinal fluid or serum specimen submitted to the state or city/county laboratory on a suspected case of arboviral disease), and 3) have an environmental surveillance system that includes mosquito surveillance to routinely monitor arboviral activity in both larval and adult mosquitoes in all parts of the jurisdiction in which there is the potential for human outbreaks of arboviral disease based on past experience.
